# Role of inflammasomes/pyroptosis and PANoptosis during fungal infection

**DOI:** 10.1371/journal.ppat.1009358

**Published:** 2021-03-18

**Authors:** Benoit Briard, R. K. Subbarao Malireddi, Thirumala-Devi Kanneganti

**Affiliations:** Department of Immunology, St. Jude Children’s Research Hospital, Memphis, Tennessee, United States of America; Vallabhbhai Patel Chest Institute, INDIA

## Introduction

The innate immune system is essential for recognizing foreign invaders and engaging a cellular and physiological response to control infection. Most human pathogenic fungi are opportunistic and take advantage of immune system defects. The 2 pathogens responsible for the majority of fungal infections in humans, *Candida albicans* and *Aspergillus fumigatus*, are no exception. One of the major components of the innate immune system that has critical roles in fighting microbial infections, including fungal infections, is the inflammasome [1,2]. Inflammasomes are multimeric protein platforms that lead to inflammatory caspase-1 activation, which in turn drives proteolytic maturation of proinflammatory cytokines IL-1β and IL-18. Processing of IL-1β and IL-18 could also be inflammasome-independent during infections due to host intrinsic proteases, and this has been extensively reviewed elsewhere [3]. Active caspase-1 also parallelly induces the cleavage and activation of gasdermin D (GSDMD), which forms pores in the plasma membrane, leading to a lytic form of inflammatory cell death called pyroptosis and facilitating the extracellular release of IL-1β and IL-18 [4,5]. To date, multiple innate sensors with the potential to assemble inflammasome complexes have been identified, including the nucleotide-binding domain and leucine-rich repeat receptors (NLRs) NLRP1, NLRC4, and NLRP3 as well as absent in melanoma 2 (AIM2) and pyrin [1].

Inflammasomes are activated when the sensor recognizes pathogen-associated molecular patterns (PAMPs) and damage-associated molecular patterns (DAMPs). In the context of fungal infection, polysaccharides, which are the major component of the fungal cell wall, serve as a large source of fungal PAMPs. Cell wall-purified zymosan and mannan were the first fungal ligands shown to activate the canonical NLRP3 inflammasome [6]. While several fungal species are now known to be capable of activating inflammasomes, including *C*. *albicans*, *A*. *fumigatus*, *Cryptococcus neoformans*, *Paracoccidioides brasiliensis*, *Malassezia* spp., and *Microsporum canis*, among others [7–13], the full range of fungal PAMPs responsible for inflammasome activation remains a key topic of study for several of these pathogens.

Growing understanding of the physiological importance of inflammasomes in antimicrobial defense has resulted in the rapid exploration of fungi-induced inflammasome activation [6,14,15]. Initial studies focused on the role of the IL-1 family cytokines IL-1β and IL-1α in restricting the fungal pathogen *C*. *albicans* during systemic infection have provided convincing evidence for the role of inflammasomes in antifungal immunity [14]. Similarly, mice lacking IL-1β and/or IL-18, the 2 inflammasome-dependent effector cytokines, are susceptible to aspergillosis [15]. Here, we focus on the molecular mechanisms of inflammasome activation and its importance for host defense in the context of the 2 most deadly fungi in humans: *C*. *albicans*, a dimorphic yeast, and *A*. *fumigatus*, a filamentous fungus.

## Candida albicans

### Molecular mechanisms of inflammasome regulation

**Fig 1:**
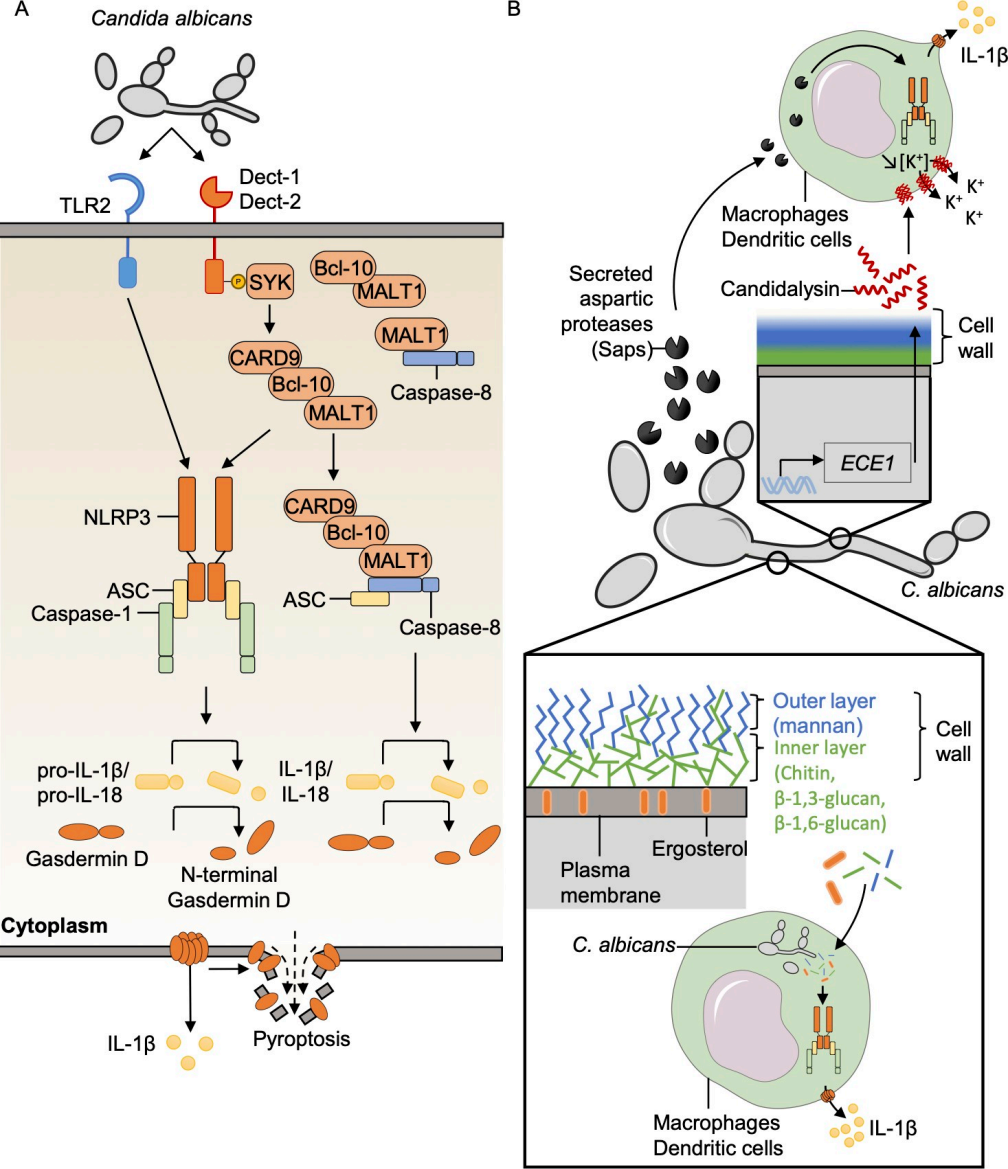
Mechanisms of *C*. *albicans*-induced inflammasome activation. (**A)** *C*. *albicans* is recognized by TLRs and Dect receptors to mediate efficient priming and inflammasome activation. The CLR pathway is activated via the adaptor protein SYK, and the downstream supramolecular complex CARD9–Bcl-10–MALT1 drives the recruitment of caspase-8/ASC and/or NLRP3/ASC/caspase-1 to cleave gasdermin D and process and release IL-1β. (**B)** PAMPs and toxins from *C*. *albicans* that can activate the inflammasome have been identified. The *C*. *albicans* Saps mediate inflammasome activation. During germination of *C*. *albicans*, *ECE1* regulates candidalysin toxin production and secretion; candidalysin forms pores in the host cell membrane and induces potassium efflux to cause canonical NLRP3 inflammasome activation. The cell wall, composed of an inner layer (Chitin/β-1,3-glucan/β-1,6-glucan) and an outer layer (mannan), participates in inflammasome activation. ASC, apoptosis-associated speck-like protein containing a caspase activation and recruitment domain; Bcl-10, B-cell lymphoma/leukemia 10; CARD9, caspase activation and recruitment domain-containing protein 9; CLR, C-type lectin receptor; Dect, Dectin; IL-1β, interleukin 1 beta; IL-18, interleukin 18; MALT1, mucosa-associated lymphoid tissue lymphoma translocation protein 1; NLRP3, nucleotide-binding domain and leucine-rich repeat family pyrin domain-containing 3; PAMP, pathogen-associated molecular pattern; Saps, secreted aspartic proteases; SYK, spleen tyrosine kinase; TLR, Toll-like receptor.

In vitro, *C. albicans* induces caspase-1 cleavage and IL-1β release in an NLRP3– and caspase-1– dependent manner in macrophages and dendritic cells (DCs) (Fig 1A) [16–19]. Fungi-induced inflammasome activation also requires the C-type lectin receptor (CLR) pathway. In particular, the Dectin-1-SYK-CARD9 signaling axis is critical to activate caspase-1, leading to the release of IL-1β and IL-18 (Fig 1A) [16,18,20–25]. Both Toll-like receptor (TLR) and CLR innate immune pathways contribute to fungal recognition and the cellular signaling necessary for priming and activation of the NLRP3 inflammasome (Fig 1A) [22]. However, priming with an exogenous ligand does not rescue inflammasome activation in *Dectin1*^*−/−*^ bone marrow-derived DCs (BMDCs), suggesting a priming-independent role for the CLR pathway in inflammasome activation during *Candida* infection [26].

**Fig 2:**
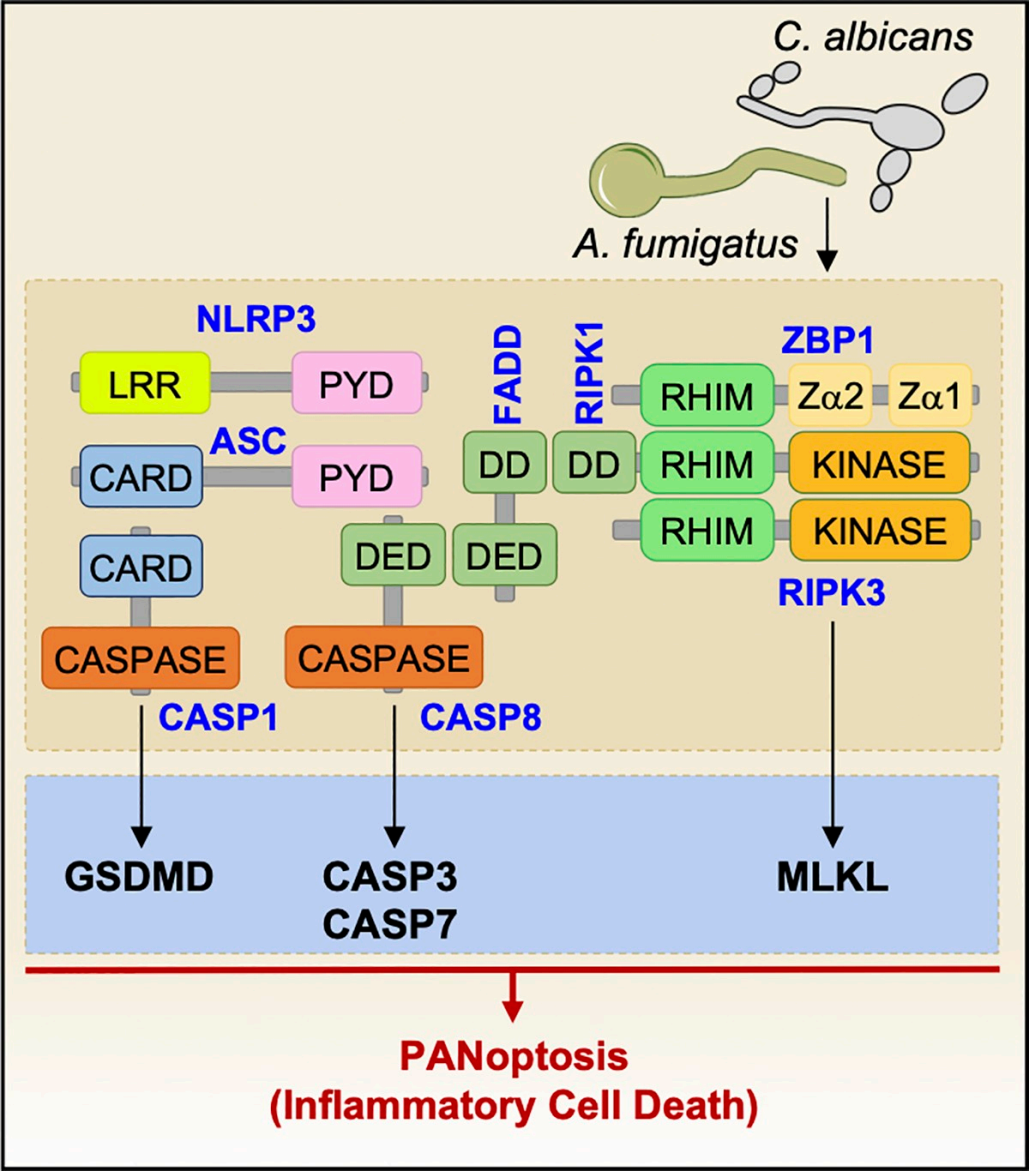
Graphical representation of PANoptosome/PANoptosis. ZBP1 senses *C*. *albicans* and *A*. *fumigatus* infection to mediate PANoptosome (ZBP1-RIPK1-RIPK3-FADD-CASP8) complex formation and drive PANoptosis, inflammatory cell death characterized by NLRP3/CASP1 activation (pyroptosis), CASP3/CASP7 activation (apoptosis), and MLKL activation (necroptosis). ASC, apoptosis-associated speck-like protein containing a caspase activation and recruitment domain; CARD, caspase activation and recruitment domain; CASP, caspase; DD, death domain; DED, death effector domain; FADD, fas-associated death domain protein; GSDMD, gasdermin D; LRR, leucine-rich repeat; MLKL, mixed lineage kinase domain-like pseudokinase; NLRP3, nucleotide-binding domain and leucine-rich repeat family pyrin domain-containing 3; PYD, pyrin domain; RHIM, receptor-interacting protein homotypic interaction motif; RIPK, receptor-interacting serine/threonine kinase; ZBP1, Z-DNA-binding protein 1.

Dectin-1 signaling during treatment with the fungal PAMP curdlan in vitro induces the formation of a Dectin-1/SYK downstream complex composed of CARD9, Bcl-10, MALT1, and caspase-8 [20]. Caspase-8 is then necessary to recruit and interact with ASC and process pro–IL-1β (Fig 1A) [20]. Moreover, before fungal recognition and inflammasome activation, MALT1 constitutively forms 2 independent complexes, one with Bcl-10 and one with caspase-8; whether these constitutive complexes have specific function requires further investigation (Fig 1A) [20]. Recently, the innate immune sensor Z-DNA binding protein 1 (ZBP1) has been shown to be a fungal sensor during *C*. *albicans* infection to activate the inflammasome and PANoptosis, a unique inflammatory programmed cell death pathway regulated by the PANoptosome complex (Fig 2) [27]. The Zα2 domain of ZBP1 is crucial for triggering the activation of cell death pathways, but the fungal ligand remains unknown [27].

Several components of *C*. *albicans* have been shown to induce inflammasome activation. The *C*. *albicans* secreted aspartic proteases (Saps), Sap2 and Sap6, induce canonical activation of the NLRP3 inflammasome (Fig 1B) [28,29]. Fungal strains lacking Saps induce significantly less neutrophil influx and IL-1β release [30]. Candidalysin, a cytolytic peptidic toxin secreted from the hyphal form of *C*. *albicans*, induces cell damage and death and canonical NLRP3 inflammasome activation [31–35]. Candidalysin forms pores in the plasma membrane of macrophages and DCs to drive potassium efflux-mediated inflammasome activation [31]. A fungal strain lacking *ECE1* (*ece1Δ/Δ*), which is defective in the production of candidalysin, causes reduced IL-1β release in human macrophages but not in murine cells [31]. However, purified candidalysin acts similarly on both human and mouse cells [31]. Endocytosis of the toxin is required to induce potassium efflux and subsequent NLRP3 inflammasome activation, but candidalysin induces pyroptosis in a caspase-1–independent manner [31]. Interestingly, the *ece1Δ/Δ* strain induces significantly less IL-1β release compared to the parental strain, which results in a failure to control the fungal burden during central nervous system infection with the mutant in mice [36].

In addition to the secreted molecules that mediate inflammasome activation, *C*. *albicans* cell wall components are also important drivers. *C*. *albicans* hyphae have been reported to contribute to inflammasome activation [17]. However, other studies showed hyphal switching is not essential [26,37]. Indeed, it has been shown that β-(1,3)-glucan present on the yeast form in the inner layer of the cell wall can activate the inflammasome (Fig 1B) [6,38,38–40], but fungal mutants deficient in glucan exposure are still able to induce pyroptosis [41]. The cell surface localization of ergosterol is also critical for pyroptosis, and the presence of *O*-mannoproteins is required for efficient exposition of ergosterol on the *C*. *albicans* surface (Fig 1B) [42]. The importance of hyphal switching remains to be fully established. Overall, *C*. *albicans* induces inflammasome activation through both secreted and cell surface molecules, which has important implications for a protective host response.

### *C*. *albicans* mediating cell death

*C*. *albicans* induces GSDMD cleavage and pyroptosis in bone marrow-derived macrophages (BMDMs) (Fig 1A) [27,38,41]. Interestingly, *C*. *albicans* induces 2 sequential types of cell death. During the initial stage of infection, the yeast predominantly drives pyroptotic cell death downstream of caspase-1 [38]. Then, a second phase of caspase-1–independent cell death occurs [38,41]. It is proposed that in 2 concomitant mechanisms, hyphae physically rupture the cell membrane while fungal growth consumes the glucose from the host cells that is necessary for them to survive, finally permitting the escape of *C*. *albicans* from macrophages [38,41,43]. Furthermore, *C*. *albicans* induces PANoptosis, characterized by caspase-1 activation (pyroptosis), caspase-8, caspase-3, and caspase-7 activation (apoptosis), and MLKL phosphorylation (necroptosis), in a ZBP1-dependent manner [27].

### Inflammasomes in host resistance to *C*. *albicans*

Mice lacking the inflammasome components NLRP3, ASC, and caspase-1 are highly susceptible to systemic candidiasis [16,18,24]. In addition to NLRP3, NLRC4 has also been shown to play antifungal roles in the stromal compartment during mucosal infection [16,44]. During vulvovaginal candidiasis, the NLRP3 inflammasome is required to control *C*. *albicans* dissemination [30,45]. However, in an oral mucosal model, NLRC4 is necessary to dampen the detrimental proinflammatory effect of NLRP3 activation. NLRC4 is activated in response to IL-22 and secretes the anti-inflammatory cytokine IL-1ra to protect against detrimental inflammation induced by NLRP3 [45]. Whether intrinsic *C*. *albicans* or microbiota pattern components regulate and activate NLRC4 during fungal mucosal infection requires further investigation. *C*. *albicans* also triggers inflammasome activation and bioactivation of IL-1β in microglial cells, which in turn promotes CXCL1-neutrophil–mediated control of the fungal infection [36]. Additionally, inflammasome activation in microglia requires the CARD9–Bcl-10–MALT1 complex downstream of C-type lectins [36,46]. Overall, it is clear that inflammasome activation plays an important role in host defense against *C*. *albicans* infection and that balancing this activation is critical to prevent hyperinflammation.

## Aspergillus fumigatus

### Molecular mechanisms of inflammasome regulation

**Fig 3:**
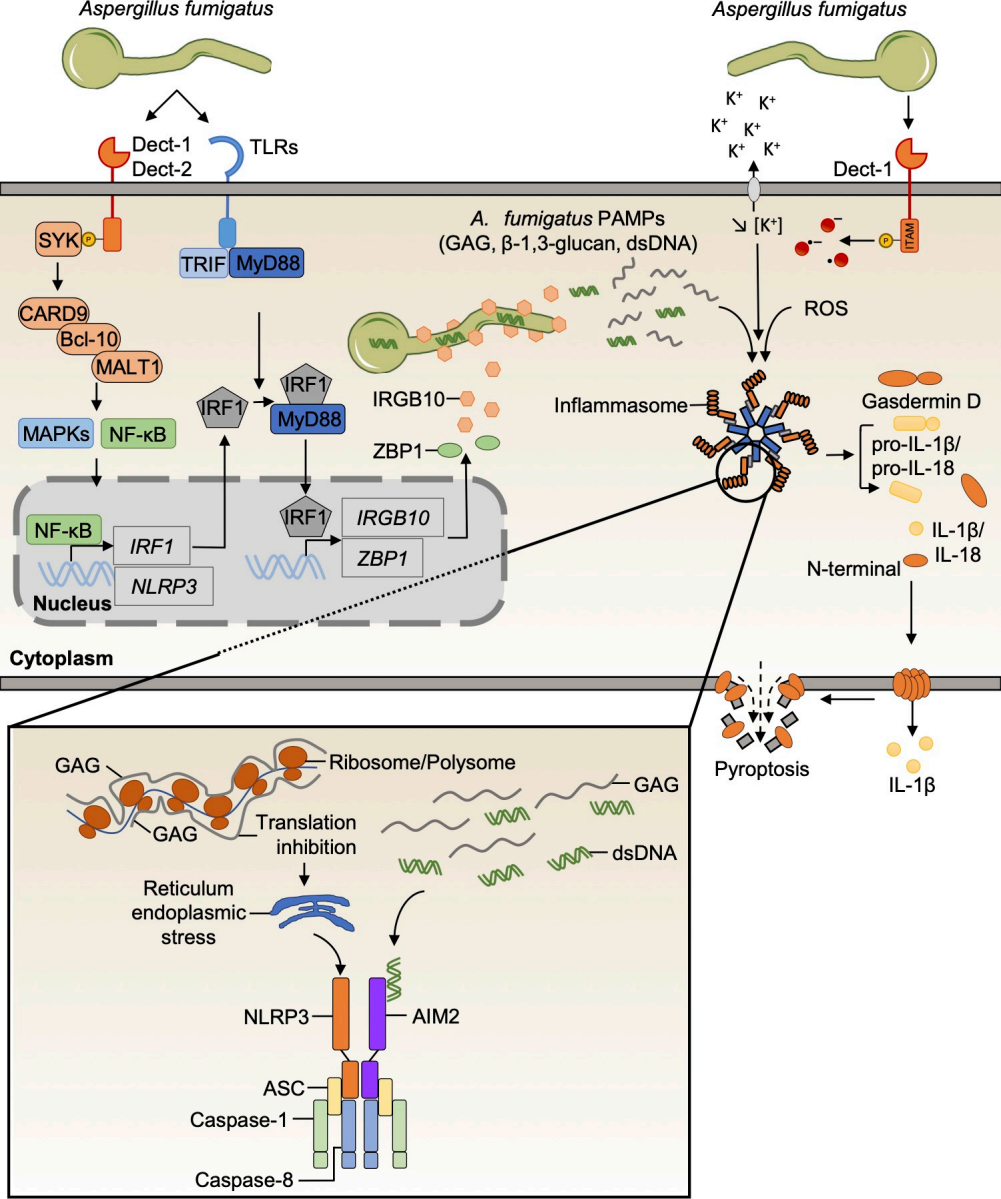
Mechanisms of *A*. *fumigatus*-induced inflammasome activation. *A*. *fumigatus* hyphae induce canonical inflammasome activation after recognition by the CLR and TLR pathways, which is dependent on ROS production and potassium efflux. SYK activation initiates the formation of a supramolecular CARD9–Bcl-10–MALT1 complex to drive MAPKs and NF-κB signaling and induce inflammasome priming. Parallelly, SYK regulates ROS production to mediate the canonical activation of the NLRP3 inflammasome. The NF-κB pathway regulates the expression of IRF1, and the TLR pathway mediates efficient activation of IRF1 after recognition of *A*. *fumigatus* via the adaptor molecules MyD88 and TRIF. IRF1 expression and activation induce IRGB10 and ZBP1 expression. IRGB10 targets the fungal cell surface and causes hyphae damage, inhibiting *A*. *fumigatus* growth and releasing GAG, β-1,3-glucan, and dsDNA to activate the inflammasome. GAG interacts with ribosomes to block translation, inducing ER stress and NLRP3 inflammasome activation, whereas *A*. *fumigatus* dsDNA is recognized by the AIM2 inflammasome. ASC, apoptosis-associated speck-like protein containing a caspase activation and recruitment domain; Bcl-10, B-cell lymphoma/leukemia 10; CARD9, caspase activation and recruitment domain-containing protein 9; CLR, C-type lectin receptor; Dect, dectin; ER, endoplasmic reticulum; GAG, galactosaminogalactan; IL-1β, interleukin 1 beta; IL-18, interleukin 18; IRGB10, immunity-related GTPase B10; IRF1, interferon regulatory factor 1; ITAM, immunoreceptor tyrosine-based activation motif; MALT1, mucosa-associated lymphoid tissue lymphoma translocation protein 1; MAPKs, mitogen activated protein kinases; NF-κB, nuclear factor kappa B; NLRP3, nucleotide-binding domain and leucine-rich repeat family pyrin domain-containing 3; MyD88, myeloid differentiation primary response 88; PAMPs, pathogen-associated molecular patterns; ROS, reactive oxygen species; SYK, spleen tyrosine kinase; TLR, Toll-like receptor; TRIF, Toll/interleukin 1 receptor domain-containing adapter-inducing interferon-β; ZBP1, Z-DNA-binding protein 1.

*A*. *fumigatus* is a filamentous fungus that triggers inflammasome activation in an NLRP3-dependent manner in macrophages and DCs [15,47]. *Aspergillus*-induced NLRP3 inflammasome activation in vitro is dependent on potassium efflux and reactive oxygen species (ROS) production, but not caspase-11 (Fig 3) [15,47]. However, unlike *C*. *albicans*, *A*. *fumigatus* requires the AIM2 receptor for an efficient inflammasome response in murine DCs [15]. Interestingly, NLRP3 and AIM2 are redundant and simultaneously present in the same inflammasome complex with the adaptor ASC, caspase-1, and also caspase-8 (Fig 3) [15,20]. Moreover, caspase-8 and FADD drive caspase-1 activation and IL-1β release in BMDCs [15].

In contrast to macrophages and DCs, in vitro, neutrophils require caspase-11 for caspase-1 activation and IL-1β release in response to *A*. *fumigatus* [15,48,49]. It will be interesting to investigate whether the caspase-11/gasdermin axis drives secondary NLRP3 inflammasome activation in neutrophils.

*A*. *fumigatus* is sensed by both TLRs and CLRs, which together orchestrate an effective innate immune response to this pathogen (Fig 3) [50]. Indeed, chemical inhibition or genetic deletion of the CLR pathway abrogates *Aspergillus*-induced caspase-1 activation and IL-1β secretion [20,40,47,49]. Activation of the CLR pathway induces the formation of a CARD9–Bcl-10–MALT1–caspase-8 complex for inflammasome activation [20,40]. Perturbation of this complex also directly affects NF-κB signaling and, hence, expression of the NLRP3 inflammasome (Fig 3) [40]. Future studies should help us understand priming-independent functions of this signalosome complex in promoting the activation of NLRP3–AIM2–ASC–caspase-1 inflammasome complexes in response to *Aspergillus* and other fungal pathogens.

The interferon pathway is also essential for inflammasome activation and particularly for the expression of the transcription factor IRF1 and IRGB10 [51,52]. Fungi-induced CLR pathways drive the expression of IRF1, and cells lacking IRF1 are defective in inflammasome activation in response to *A*. *fumigatus* (Fig 3) [40,51,53]. IRF1 is activated during *A*. *fumigatus* recognition through the TLR pathway (Fig 3) [40,54]. During *A*. *fumigatus* infection, the immunity-related GTPase IRGB10 targets the fungal cell wall to facilitate the release of PAMPs that, in turn, drive inflammasome activation (Fig 3) [40]. Similarly, ZBP1, an IRF1-dependent protein, is also required for *A*. *fumigatus*-mediated inflammasome activation (Figs 2 and 3) [27]. However, the *A*. *fumigatus* ligand recognized by the sensor ZBP1 is unknown and requires further studies.

Galactosaminogalactan (GAG) was recently identified as a specific *A*. *fumigatus* PAMP that activates the NLRP3 inflammasome [55]. GAG is a polysaccharide present at the surface of the *A*. *fumigatus* cell wall and is secreted [56]. Mechanistically, the phagocytosed conidia grow and synthesize GAG. The galactosamine moiety then interacts with ribosomes by charge–charge interactions [55]. GAG immobilizes the ribosome and polysome to inhibit translation, which induces endoplasmic reticulum stress and triggers NLRP3 inflammasome activation [55]. β-(1,3)-glucan in the cytosol has also been shown to directly activate the inflammasome in macrophages [40], but the exact mechanism is unclear. Future studies should address whether there is a general mechanism for polysaccharide sensing and what role, if any, the biochemical properties of the specific polymers play in inducing inflammasome activation and/or priming. Further studies will allow for the discovery of new cytosolic sensors and inflammasome activation mechanisms.

### *Aspergillus* mediating cell death

Pyroptosis has not been well characterized during *A*. *fumigatus* infection. The cleavage of GSDMD was recently identified in macrophages during *A*. *fumigatus* infection (Fig 3). Additionally, similar to what has been observed with *C*. *albicans*, *A*. *fumigatus* induces PANoptosis in a ZBP1-dependent manner (Fig 2) [27]. It has previously been assumed that hyphal growth induces cell death through physical damage, but it was recently demonstrated that *A*. *fumigatus* hyphae form a network through the bronchial epithelium which forms an actin gate and permits hyphae to pierce the plasma membrane without inducing cell death [57]. It will be interesting to determine if similar mechanisms exist in *Aspergillus*-infected macrophages and DCs.

### Inflammasomes in host resistance to *A*. *fumigatus*

*A*. *fumigatus* often acts as a pulmonary pathogen which infects immunocompromised patients and induces varying pathologies, the most grave being invasive pulmonary aspergillosis (IPA) [58]. Loss of both NLRP3 and AIM2 inflammasome components in mice results in susceptibility to IPA [15,48]. Both inflammasomes are required in hematopoietic and stromal compartments to protect against fungal infection [15]. Although NLRC4 was shown to be protective in a mucosal model of *C*. *albicans* infection [44,45], its role during IPA is not known. IRGB10-deficient mice, which are defective in caspase-1 activation and IL-1β release, were also shown to be susceptible to infection [40]. Furthermore, *A*. *fumigatus* that overproduce GAG and enhance inflammasome activation elicit more protective responses from the host and improve survival compared with wild-type *A*. *fumigatus*, while a fungal strain that is deficient in GAG has increased virulence during systemic infection [55]. Similarly, in a model of colitis, an inflammatory disease where inflammasome activation preserves colon homeostasis, GAG treatment protects mice by inducing inflammasome activation and IL-18 release [55,59]. Collectively, these findings highlight the importance of GAG in inflammasome activation and in host protection during *A*. *fumigatus* infection.

Beyond canonical inflammasome activation, mice lacking caspase-11 do not control the fungal burden in an *Aspergillus* keratitis infection model or in IPA [48,49]. Moreover, mice lacking both caspase-1 and caspase-11 are more susceptible than caspase-1–deficient mice. Caspase-11 is required for inflammasome activation only in neutrophils in response to *A*. *fumigatus* [48,49]. Since neutrophils are essential to fight aspergillosis, it is important to further understand the role of caspase-11 in inflammasome-dependent and inflammasome-independent functions of neutrophils. Whether *A*. *fumigatus* activates caspase-11 through an LPS-independent mechanism or via host microbiome–mediated cell death needs to be explored further.

## Discussion

The central role of inflammasomes in health and disease has triggered enormous interest in this area of research over the last decade and continues to entice scientists. However, study of the role and regulation of inflammasomes in fungal infections is still in its infancy, and several important questions remain unanswered. These unexplored areas include the role of interferons (IFNs) in inflammasome regulation during fungal infection. Type I IFN (IFN-I) acts as a central regulator of inflammasome activation in bacterial and viral infections [60], but its role in fungal infections is poorly understood and currently controversial [61]. The IFN-I receptor IFNAR1 is required for inflammasome activation in neutrophils infected with *A*. *fumigatus* [49], but no evidence has been found to support this function in macrophages or DCs. Additionally, during *C*. *albicans* infection, Saps mediate IFN-I secretion for caspase-11 activation and cooperate with NLRP3 inflammasome activation [62]. However, IFN-I has also been shown to inhibit inflammasome activation and IL-1β production in response to *C*. *albicans* [63]. It is not clear why opposing mechanism are observed. Is this phenomenon specific to *C*. *albicans* or general to a range of fungal pathogens? Is IFN-I required for caspase-11–mediated NLRP3 inflammasome activation during fungal infection? Further investigation into the role of IFNs in the regulation of inflammasomes during fungal infection is necessary.

The inflammasomes induced by fungi require caspase-8 in addition to caspase-1. Caspase-8 is recruited to the NLRP3 inflammasome complex and is necessary for pro–IL-1β processing [15,20,64]. Indeed, it has been observed that caspase-8 can process the pro-form of IL-1β independent of caspase-1 in a dectin-1–dependent manner [20]. Recently, the link between pyroptosis, apoptosis, and necroptosis (PANoptosis) has been highlighted [27,65]. It will be interesting to further study the supramolecular complex, the PANoptosome, formed in response to fungi to understand whether it is dependent on the C-type lectin pathway.

Interestingly, B lymphocytes (B cells), T cells, and T regulatory cells have a CARD11, Bcl-10, MALT1 (CBM) signalosome complex downstream of the B cell receptor (BCR) and T-cell receptor (TCR) that is similar to the complex formed in the CLR pathway necessary for fungal recognition and inflammasome activation [66]. Mutations causing inhibition or aberrant activation of the human CBM signalosome complex are associated with primary immunodeficiency diseases, cancers, and infectious diseases [66]. Because B cells and T cells express all of the inflammasome components [67–69], it will be interesting to study the interaction between CBM complex and inflammasome components in lymphocytes. These findings may complement the mechanisms of fungi-induced inflammasome activation observed in myeloid cells.

Additionally, recognition of bacterial and viral ligands in the cytosol by the inflammasome-forming innate sensors is well studied [60]; however, the fungal ligands triggering inflammasome activation are poorly characterized to date. β-(1,3)-glucan and mannan with ATP treatment are known to activate the canonical NLRP3 inflammasome, and recently, the presence of GAG or β-(1,3)-glucan in the cytosol was shown to directly activate NLRP3 in macrophages [6,40,55]. The mechanism of NLRP3 inflammasome activation has been elucidated for GAG but remains to be discovered for other fungal PAMPs.

Fungal polysaccharide-mediated inflammasome control has been found to be beneficial in multiple disease models. For example, GAG-mediated inflammasome activation is protective in a murine colitis model [55]. Additionally, β-(1,3)-glucan mediates reprogramming and training of innate immune cells [70], and β-(1,3)-glucan–reprogrammed macrophages have impaired canonical NLRP3 inflammasome activation [71]. When macrophages from patients with cryopyrin-associated autoinflammatory syndrome are treated with β-(1,3)-glucan, they have reduced caspase-1 activation and IL-1β release compared with untreated macrophages [71], which could be therapeutically beneficial for these patients. These finding should open new perspectives in studies focused on the ability of fungal polysaccharides to modulate inflammasome activation as potential therapeutic strategies. Indeed, the inflammasome can have both beneficial and detrimental roles, and the fine-tuning of its activity is crucial during disease to activate protective inflammatory cascades during *C*. *albicans* or *A*. *fumigatus* infections while avoiding detrimental inflammation [45,72,73]. Targeting inflammasome activity could have important clinical applications to enhance the immune response during fungal infection or to reduce its effects when needed. The clinical relevance of inflammasomes during fungal infection is still undercharacterized, and further clinical studies should help to decipher this role.

The role of inflammasomes during fungal infection is crucial in host resistance. Improving our understanding of this specific regulation may lead to the discovery of novel fungi-specific mechanisms distinct from bacteria or viruses and new drug targets to treat fungal infections, which remain difficult to eradicate.

### Disclaimer

The content is solely the responsibility of the authors and does not necessarily represent the official views of the National Institutes of Health.
